# PrEP-related stigma and PrEP use among gay, bisexual and other men who have sex with men in Ontario and British Columbia, Canada

**DOI:** 10.1186/s12981-022-00473-0

**Published:** 2022-10-27

**Authors:** Oscar Javier Pico-Espinosa, Mark Hull, Paul MacPherson, Daniel Grace, Mark Gaspar, Nathan Lachowsky, Saira Mohammed, James Demers, Marshall Kilduff, Robinson Truong, Darrell H. S. Tan

**Affiliations:** 1 St Michael’s Hospital, Toronto, ON Canada; 2 BC Centre for Excellence in HIV/AIDS, Vancouver, BC Canada; 3 University of Ottawa, Ottawa, ON Canada; 4 University of Toronto, Toronto, ON Canada; 5 University of Victoria, Victoria, BC Canada; 6 MAX Ottawa, Ottawa, ON Canada; 7 AVI Health and Community Services, Victoria, BC Canada

**Keywords:** HIV pre-exposure prophylaxis, Social stigma, Health promotion, Retention in care, Health services accessibility

## Abstract

**Background:**

We aimed to explore the association between PrEP-related stereotypes and perceived disapproval (hereafter PrEP-related stigma), and PrEP use.

**Methods:**

We used data from a cross-sectional online survey among adult gay, bisexual, other men who have sex with men in Ontario and British Columbia, Canada. Participants were recruited 2019–2020 in-person from sexual health clinics and outreach programs, and online through dating mobile applications and websites. We used logistic regression models to explore the relationship between PrEP-related stigma and: 1-being a ‘never’ versus ‘current’ PrEP user, and 2-being a ‘former’ versus ‘current’ user.

**Results:**

The median age of the sample was 32 (Q1-Q3 = 27–40), most were white born in Canada (48%), 45% had never used PrEP, 16% were former PrEP users and 39% were current PrEP users. Of 1527 individuals who started the survey, 1190 participants answered questions about PrEP-related stigma: 254 (21.3%) were classified as having low level of PrEP-related stigma, 776 (65.2%) intermediate, and 160 (13.5%) high. No significant association was found when never PrEP users and current PrEP users were compared: adjusted OR = 1.44 (95%-CI: 0.8–2.5). High PrEP-related stigma was positively associated with being a former PrEP user compared to being a current PrEP user: adjusted OR = 2.5 (95%-CI: 1.3–4.9).

**Conclusion:**

PrEP-related stigma is associated with not using PrEP, particularly with PrEP discontinuation. Our findings indicate that stigma persists as a barrier to PrEP use.

**Supplementary Information:**

The online version contains supplementary material available at 10.1186/s12981-022-00473-0.

## Introduction

Despite advances in the prevention, diagnosis and treatment of HIV, HIV and other STBBIs remain a significant health concern in Canada [1]. The national diagnosis rate was 5.6 per 100,000 population in 2019, and gay, bisexual and other men who have sex with men (GBM) remain the largest proportion of new HIV diagnoses [1]. HIV pre-exposure prophylaxis (PrEP) using tenofovir disoproxil fumarate and emtricitabine (TDF-FTC) is an effective biomedical intervention to reduce HIV risk [2–5] that has been approved for use in Canada since 2016. Efficient PrEP rollout is associated with a decline in number of new HIV cases [6]. However, various barriers exist at the individual, interpersonal, community and organizational level [7]. One of such barriers is stigma [7]. Stigma is “a social process or related personal experience characterized by exclusion, rejection, blame, or devaluation that results from experience or reasonable anticipation of an adverse social judgment about a person or group identified with a particular health problem” [8]. Individuals with a certain characteristic are seen by others as being of lower social value or a less desirable person [9]. As a consequence, such individuals encounter prejudice and discrimination [10].

Stigma can be experienced, perceived, anticipated, enacted and internalized [11]. Being stigmatized can be associated with poor health because stigmatized individuals become at risk of having less resources such as beneficial social connections, prestige and power; being socially isolated and restricted to a circle of individuals sharing the same experiences of stigma; internalizing negative perceptions and/or redirection of such negative perceptions towards individuals of the same group; adopting unhealthy coping behaviors; and increased stress [12].

PrEP can be seen as a tool against stigma surrounding HIV and sexuality [11, 13]. PrEP can potentially reduce anxiety related to sexual encounters, including with partners living with HIV, and subsequently increase sexual pleasure, an often unacknowledged benefit of PrEP [14, 15]. Despite this, PrEP users can be the object of stigma from themselves, friends, family, sexual partners, healthcare providers and society at large, as they can be stereotyped as promiscuous, irresponsible and immoral [11, 13, 16, 17].

Some data from developed countries indicate that, as PrEP use has become more normalized, PrEP-related stigma may be decreasing [18]. However, stigma is often cited as a major barrier to PrEP uptake and adherence [19–22]. A survey from 2018 among GBM and transgender women (TGW) in the United States showed that believing that PrEP is for people who are promiscuous was strongly associated with lack of interest in using PrEP [23]. Some individuals may avoid seeking or staying on PrEP as a consequence of anticipated or experienced stigmatization [14]. Furthermore, stigma overlaps with HIV-related stigma, in a way that fear of HIV may decrease PrEP uptake as it prevents individuals from openly talking about HIV [24]; similarly, it overlaps with homophobia, which is important because individuals might decline PrEP or stop using it because they may not want to be associated with a particular sexual orientation [24].

To facilitate seeking and continuing use of PrEP, positive messaging from others who share the same identity (GBM, TGW), society-at-large, and institutions (e.g. education, medical) is necessary to encourage individuals to acknowledge and embrace their desire to practice safer, more intimate and more enjoyable sex [17]. Therefore, more efforts to characterize PrEP-related stigma and how it influences PrEP uptake are warranted. We aimed to explore whether there is an association between perceptions of PrEP-related stigma and PrEP use in a sample of GBM and TGW in Ontario and British Columbia, Canada.

## Methods

### Study population

We used data from a cross-sectional survey from the PrEP implementation project (PRIMP), a multicomponent study with the objective of scaling up PrEP uptake in urban Ontario and British Columbia, Canada.[25] GBM and TGW were recruited between July 2019 and August 2020 through advertisements on dating apps, social media and sexual health clinics in each province’s largest metropolitan areas: Toronto, Ottawa, Hamilton, Vancouver and Victoria. Additional inclusion criteria were being at least 19 years of age, not living with HIV, reporting recent sex with a man and being able to communicate in English. Staff and volunteers of partner community organizations were not eligible. Participants self-completed an electronic questionnaire about demographics, sexual health, attitudes towards PrEP, and knowledge of/experience with PrEP. Participants received a $10 CAD gift card for their participation.

### Variables and definitions

The independent variable, PrEP-related stigma, was constructed based on the answers to the following three four-point Likert-type questions: (a) “*I think people who take PrEP are promiscuous*”, (b) “*Other people think people who take PrEP are promiscuous*” and (c) “*I think people who take PrEP are responsible*”. We used items (a) and (b) to capture PrEP-related stigma because considering PrEP users to be “promiscuous” is among the most common ways that people deem PrEP users to be of lower social value; including the two items together allowed us to consider respondents’ personal and broader social perceptions about PrEP-related stigma, respectively. We included item (c) because associating PrEP with being “responsible” represents an opposing view, in which PrEP use is conceived as desirable and of greater social value [13]. Points were assigned to each answer in questions “a” and “b” as follows: *strongly disagree*, 1 point; *disagree*, 2 points; *agree*, 3 points and; *strongly agree*, 4 points. Question “c” was reverse scored. We summed up the individual scores; the minimum total score was 3 and the maximum was 12. Three levels of perceived PrEP-related stigma were defined based on the total score: low PrEP-related stigma, 3–5 points; intermediate PrEP-related stigma, 6–8 points; and high PrEP-related stigma, 9–12 points. These cutoffs were chosen based on the distribution of the scores (normally distributed) among the study sample, with the values of the intermediate PrEP-related stigma category laying between − 1 and + 1 standard deviations from the mean (see supplement). The dependent variable was PrEP use: never PrEP use, former PrEP use and current PrEP use as reported by the participants at the time of the survey.

Potential confounders were chosen based on existing literature [7, 20, 21, 23, 26–30] and discussions within the team. We selected the following variables: (1) sociocultural background (measured as a combination of the variables country of birth: born in Canada vs. born abroad, and ethnicity: White, Black, Latinx, East Asian, Indigenous and others (this variable was constructed to better represent the sociocultural diversity of the study setting); (2) level of education; (3) age in years; (4) disclosure of sexual orientation; (5) proportion of gay, bisexual and queer (GBQ) people in their social network; (6) endorsing the statement “condoms are the only truly effective form of HIV prevention; (7) being worried about getting STIs; (8) number of male sexual partners in the past six months; and (9) clinical indication for PrEP. The latter was defined as having had condomless anal sex in the past six months plus any of the following: an HIV-incidence risk indicator score (HIRI-MSM) [31] ≥ 11, history of infectious syphilis, rectal gonorrhea or rectal chlamydia and use of HIV non-occupational post-exposure prophylaxis at least twice, according to Canadian guidelines [4]. We did not collect information on whether the participant had an ongoing relationship with an HIV-positive person with transmissible HIV, which is an additional albeit uncommon clinical indication for PrEP suggested in the guidelines [4].

### Analyses

We present proportions for categorical variables, and medians with interquartile ranges for continuous variables. Differences between groups were tested with Chi square or Fisher’s exact tests for categorical variables, and with Kruskal-Wallis H tests for continuous non-normally distributed variables. We explored the relation between the answers to the individual stigma-related questions and PrEP use by calculating the proportions and using Chi square tests. The association between the level of PrEP-related stigma and PrEP use was calculated in an available case analysis using logistic regressions. Never PrEP users and former PrEP users were compared against current PrEP users separately. We used a directed acyclic graph to indicate the relation between potential confounders and the dependent and independent variables to select sub-sets of confounders based on the theoretical pathways between the independent and dependent variable (Supplement 1). These pathways were based on the authors’ clinical reasoning as well as published literature [28, 32–34]. Next, each potential confounder was added to the bivariate model one at a time and the change in the Beta coefficient of the independent variable was recorded. Separate models were generated by adding potential confounders, one at a time, starting with the one that resulted in a higher change in the Beta coefficient of the independent variable. The Likelihood ratio test was used to test for differences (p > 0.05) between these nested models, and AIC was used to compare non-nested models. We used the variance inflation factor (cutoff of 10) to test for collinearity. We used STATA 16 for the analyses [35].

## Results

Of a total of 1527 who initiated the questionnaire, 1190 individuals answered the questions about PrEP-related stigma; 254 (21.3%) were classified as having low perceived PrEP-related stigma, 776 (65.2%) as intermediate and 160 (13.5%) as high. Respondents with missing information on PrEP stigma were older (median age 34, Q1-Q3 = 28–45, p = 0.018) and had less years of formal education (proportion reporting high school or less was 18% vs. 11% among those with complete information, p = 0.025). Of the 1190, six participants did not provide information about their PrEP use (four were in the low stigma group and two in the intermediate stigma group). The median age was 32 years (Q1, Q3: 27, 40). Participants in the high level PrEP-related stigma group were more likely to be White and born in Canada (60%, compared with 46% in the intermediate and 49% in the low PrEP-related stigma categories); to report more often having a college degree (40%, compared to 31% in the intermediate and 24% in the low PrEP-related stigma categories) and less often a Bachelor’s degree (33%, compared with 38% in the intermediate and 47% in the low PrEP-related stigma categories) and to report a yearly income between 40.000 and 60.000 CAD (44%, compared with 27% and 32% in the medium and low level PrEP-related stigma categories respectively) (Table 1).

**Table 1:** Sociodemographic characteristics of the study population

			Stigma level						
	**All participants**		**Low**		**Intermediate**		**High**		**p-value**
**Age (years), median(Q1,Q3)**	32 (27–40)		30 (26–40)		32 (27–40)		32 (26–41)		0.838*
Total	1182		253		770		159		
**Sociocultural background**									
White born in Canada	575	48%	125	49%	354	46%	96	60%	0.01
White born abroad	97	8%	26	10%	61	8%	10	6%	
Black born in Canada	70	6%	12	5%	52	7%	6	4%	
Black born abroad	16	1%	8	3%	8	1%	0	0%	
East Asian born in Canada	35	3%	4	2%	30	4%	1	1%	
East Asian born abroad	55	5%	9	4%	42	5%	4	3%	
Latinx born in Canada	43	4%	7	3%	30	4%	6	4%	
Latinx born abroad	70	6%	13	5%	53	7%	4	3%	
Other born in Canada	79	7%	20	8%	45	6%	14	9%	
Other born abroad	105	9%	24	9%	67	9%	14	9%	
Indigenous people of Canada	41	3%	6	2%	31	4%	4	3%	
Total	1,186		254		773		159		
**Education**									
High school or less	125	11%	21	8%	86	11%	18	11%	0.009
College/technical	367	31%	61	24%	242	31%	64	40%	
Bachelor’s	464	39%	119	47%	292	38%	53	33%	
Postgraduate	224	19%	51	20%	149	19%	24	15%	
Total	1,180		252		769		159		
**Annual income (CAD)**									
20.000 or less	107	9%	23	9%	74	10%	10	6%	0.006
20.001-40.000	247	21%	50	20%	174	23%	23	15%	
40.001-60.000	357	30%	82	32%	205	27%	70	44%	
60.001-80.000	176	15%	31	12%	128	17%	17	11%	
More than 80.000	228	19%	51	20%	147	19%	30	19%	
Prefers not to answer	67	6%	16	6%	43	6%	8	5%	
Total	1,182		253		771		158		
**Gender**									
Man	1,146	96%	251	99%	743	96%	152	95%	0.005**
Woman	23	2%	0	0%	15	2%	8	5%	
Two-Spirit	4	1%	2	1%	2	1%	0	0%	
Gender Fluid	13	1%	1	1%	12	2%	0	0%	
Prefers not to answer	2	1%	0	0%	2	1%	0	0%	
Total	1,188		254		774		160		
**Relationship status**									
No regular partner	620	52%	130	51%	398	52%	92	58%	0.719
Open relationship	426	36%	94	37%	279	36%	53	33%	
Closed relationship	105	9%	23	9%	73	9%	9	6%	
Prefers not to answer	35	3%	7	3%	23	3%	5	3%	
Total	1,186		254		773		159		
**Proportion of GBQ in social network**									
≤ 25%	384	33%	72	29%	265	35%	47	30%	0.405
26–50%	328	28%	71	28%	206	27%	51	33%	
51–75%	274	24%	68	27%	170	22%	36	23%	
> 75%	175	15%	39	16%	115	15%	21	14%	
Total	1161		250		756		155		
**Disclosed sexual orientation**									
Not Out at all	52	4%	13	5%	29	4%	10	6%	< 0.001
Out to a few	255	21%	35	14%	182	23%	38	24%	
Out to half	171	14%	23	9%	109	14%	39	25%	
Out to most	215	18%	55	22%	132	17%	28	18%	
Out to all	483	41%	127	50%	312	40%	44	28%	
Prefers not to answer	13	1%	1	1%	12	2%	0	0%	
Total	1189		254		776		159		

*Kruskall-Wallis H test. **Fisher’s exact test

Participants with high perceived PrEP-related stigma were less likely to be fully open about their sexual orientation, more likely to meet clinical criteria for PrEP and more likely to agree with the statement “condoms are the only truly effective form of HIV prevention” (Tables 1 and 2).

While similar proportions of participants in the high, median and low stigma groups had never used PrEP (41%, 46% and 44% respectively), participants reporting high stigma were more likely to be former PrEP users (29% vs 13% and 14% respectively) and less likely to be current PrEP users (30% vs 40% and 44% respectively) (Table 2).

**Table 2:** PrEP-related stigma and PrEP use, other attitudes related to PrEP and number of sex partners

	PrEP-related stigma								
	Total		Low		Intermediate		High		
	n	%	n	%	n	%	n	%	
**PrEP use**									
Never user	531	45%	109	44%	356	46%	66	41%	< 0.001
Former user	189	16%	32	13%	111	14%	46	29%	
Current user	464	39%	109	44%	307	40%	48	30%	
Total	1184		250		774		160		
**PrEP regimen**									
Daily	413	66%	98	71%	265	68%	50	54%	0.001
On-demand	103	17%	16	11%	58	15%	29	31%	
Intermittently	103	17%	23	17%	66	17%	14	15%	
Total	619		137		389		93		
**PrEP indicated**									
No	343	32%	81	34%	235	34%	27	18%	0.001
Yes	738	68%	154	66%	465	66%	119	82%	
Total	1081		235		700		146		
**“Only condoms prevent HIV”** ^**a**^									
Strongly Disagree	234	20%	76	30%	136	18%	22	14%	< 0.001
Disagree	450	38%	92	37%	303	40%	55	36%	
Agree	299	25%	44	18%	206	27%	49	31%	
Strongly Agree	193	16%	39	16%	121	16%	33	21%	
Total	1176		251		766		159		
**“I am worried about STIs”** ^**b**^									
Strongly Disagree	40	3%	10	4%	21	3%	9	6%	0.012
Disagree	187	16%	45	18%	124	16%	18	12%	
Agree	539	46%	104	41%	374	49%	61	39%	
Strongly Agree	406	35%	94	37%	244	32%	68	44%	
Total	1172		253		763		156		
**Male partners past 6 m, median(Q1,Q3)**	3(1–6)		3 (1–6)		3(1–6)		3(2–9)		0.172
Total	1010		208		664		138		

^a^”Condoms are the only truly effective form of HIV prevention”. ^b^”I am worried about getting STIs like gonorrhea, chlamydia & syphilis”

We observed that 43% of former PrEP users agreed or strongly agreed with the statement “I think people who take PrEP are promiscuous”, while 35% of never PrEP users and 29% of current PrEP users agreed. Likewise, 65% of never PrEP users and 71% of former PrEP users agreed with the statement “Other people think people who take PrEP are promiscuous” in comparison with 75% of the current PrEP users. Last, 15% of former PrEP users and 12% of never PrEP users disagreed or strongly disagreed with the statement “I think people who take PrEP are responsible”, as opposed to 6% of current PrEP users (Supplement Table 1).

In multivariable models adjusting for number of sexual partners, PrEP indication and proportion of GBQ people in their social network, perceived PrEP-related stigma was not significantly associated with someone being a never PrEP user as compared with being a current PrEP user. However, high level PrEP-related stigma was positively associated with being a former PrEP user: OR 2.5, 95% CI: 1.3–4.9 after adjusting for number of sexual partners and agreeing with the statement “condoms are the only truly effective form of HIV prevention” (Table 3).

**Table 3:** Multivariable logistic regression analysis of being a never PrEP user compared to being a current PrEP user

Level of PrEP related stigma	OR	95%CI	p-value	aOR	95%CI	p-value
Never PrEP use vs. Current PrEP use* (n = 759)						
Low (referent)	1			1		
Intermediate	1.07	0.75–1.52	0.721	1.04	0.71–1.53	0.826
High	0.97	0.57–1.63	0.900	1.44	0.81–2.54	0.216
**Former PrEP use vs. Current PrEP use**† (n = 540)						
Low (referent)	1			1		
Intermediate	1.05	0.65–1.72	0.825	0.9	0.54–1.51	0.692
High	2.54	1.39–4.64	0.003	2.53	1.31–4.90	0.006

CI: confidence interval. aOR: adjusted odds ratio. *Adjusted for: Number of sexual partners, PrEP indication and size of gay/bisexual/queer social network. † Adjusted for: Number of sexual partners and agreeing with the statement “Condoms are the only truly effective form of HIV prevention”

## Discussion

In this cross-sectional study, we found an association between PrEP-related stigma and PrEP use. Former PrEP users had 2.5 greater odds compared with current PrEP users to have high level of PrEP-related stigma, after adjusting for confounding variables (number of sexual partners and agreeing with the statement “condoms are the only truly effective form of HIV prevention). Approximately one out of seven participants were classified into the high level PrEP-stigma group. We have centered our interpretation of findings on the estimated association between PrEP-related stigma and PrEP use. However, we acknowledge that many factors, including background, level of education, PrEP regimen used, among others, which were not explored in depth in the present study, might have an impact on PrEP use.

Our findings add to previous literature because they provide quantitative data to suggest that stigma may contribute to PrEP discontinuation. It has been estimated that half of PrEP users discontinue PrEP after 6–12 months [36]. PrEP discontinuation not due to decreased risk of HIV infection is a concern based on reports of higher seroconversion rates among former PrEP users [37]. In addition to financial, demographic and organizational barriers associated with PrEP discontinuation, PrEP-related stigma has been reported as a barrier to PrEP persistence in qualitative studies, which highlight that users may stop PrEP due to anticipated stigma from potential sex partners, new romantic partners, family or social network [17, 38]. In addition, some PrEP users experience judgement for not using condoms or are stereotyped as irresponsible, [17] which correlate with our findings. Importantly, our findings suggest that former users themselves think PrEP users are promiscuous, but are equally likely as current users to perceive others feeling this way. Our findings indicate that experiences while on PrEP shape individuals’ views on PrEP and their decision on whether to continue using it. However, it is unknown to what extent stigma is influenced by interactions with sexual partners, families and friends, or with healthcare providers. Nonetheless, providers should be aware of how stigma may impact decisions regarding continuing PrEP and perhaps using in the future. Therefore, they should consider discussing concerns or experiences during follow-ups in addition to the regular blood work, STI testing and prescription renewal.

Other authors have studied strategies to address stigma, mostly derived from HIV-related stigma interventions. Such strategies can be at the individual or at the structural/organizational level, including: individual-focused information dissemination, empathy induction, counselling and cognitive behavioral therapy at the individual level and; gay-affirming, school-based interventions and resiliency-focused social marketing campaigns at the structural level [39]. In addition, PrEP-related stigma can be addressed during the implementation of interventions by being inclusive without targeting stigmatized groups and, strategies could be integrated into the regular healthcare system as a routine [16]. More research is needed both to understand the impact of stigma on PrEP uptake and retention, and to generate strategies to address PrEP-related stigma.

Two other recent studies have assessed PrEP-related stigma using quantitative methods. One study, among heterosexual women in US, utilized two subscales, one to measure PrEP-user stereotypes and the other one to measure PrEP disapproval by others. PrEP-related stigma was associated with feeling less comfortable discussing PrEP with a provider and less intention to use PrEP [40]. Another study validated a scale that covers shame regarding PrEP use, character judgements of people on PrEP, and perceived social support for taking PrEP, across the anticipated, internal and experienced stigma domains. They too found an association between stigma and less willingness to take PrEP [41]. In our study, we did not use a validated instrument to measure stigma, as there were no available instruments at the time of designing the survey. In addition, our questions cover mostly perceived stigma; we did not explicitly explore internalized, anticipated or experienced stigma; and only measured stigma at the individual level. However, we measured more than one component: PrEP stereotypes and perceived disapproval by others and, our findings are in line with the cited quantitative studies. In addition, although there are various ways of measuring how the construct stigma is felt or expressed by individuals, the different dimensions or types of stigma overlap [42]. Therefore, we would likely see comparable results had we included other aspects of stigma.

### Limitations

The principal threat to the validity of our results is that we did not use a validated scale to measure PrEP-related stigma, because to our knowledge, no such scale was available when the survey was designed. Instead, we constructed a metric based on available information. We based our categorization on the distribution of the responses and the cutoffs for the scores that we use correlate with the responses to individual questions; for instance, a participant in the low-level PrEP-related stigma (scores between 3 and 5) category would have disagreed with all the stigma statements or at most, agreed with one but strongly disagreed with the other two. However, future studies could consider using validated tools such as Siegler’s scale [41]. Second, given the cross-sectional design of the study, we cannot establish whether PrEP use precedes or follows PrEP-related stigma. Stigma could precede PrEP use as stigma might build on past experiences and become part of one’s view of the world, while PrEP use can be intermittent or seasonal [36]. The opposite could also be true: PrEP use may trigger stigma surrounding sexuality or make it evident [13]. Longitudinal designs are warranted to overcome this limitation. Third, we acknowledge the possible information bias in this study. It is possible that current PrEP users did not fully disclose negative attitudes toward PrEP as this would imply a conflict between their beliefs and behaviors. If that is the case, it would decrease the strength of the association between stigma and PrEP use. Fourth, another potential bias in our study is selection bias, as highly educated, more health-conscious persons may have been more likely to participate in the survey. However, the risk of such a bias was minimized as we considered various potential confounders, increasing the generalizability of our findings. In addition, we used various recruitment strategies (onsite, through mobile applications, through social media and local networks) to establish a more diverse study population. Last, residual and unmeasured confounding cannot be excluded. Nonetheless, we considered available potential confounders and tested them in our model. Furthermore, the procedure we used to select the final set of confounders in our model did not modify the main conclusion of our study.

## Conclusion

PrEP-related stigma may be associated with PrEP discontinuation. We did not observe a significant association between stigma and being a never PrEP user versus being a current PrEP user. However, we don’t rule out the presence of other dimensions or forms of stigma among never PrEP users. Our findings indicate that stigma persists and should not be overlooked as a barrier to PrEP uptake and persistence. Strategies addressing PrEP-related stigma among potential and current PrEP users are warranted.

## Electronic supplementary material

Below is the link to the electronic supplementary material.

Supplementary Figures and Tables.

## Data Availability

(data transparency): data is confidential.

## Code availability

STATA codes and modeling log are available for review upon request.
