# Supplementary material for: PrEP-related stigma and PrEP use among gay, bisexual and other men who have sex with men in Ontario and British Columbia, Canada
Source: AIDS Res Ther. 2022 Oct 27;19:49. doi: 10.1186/s12981-022-00473-0 (PMC9615170; doi:10.1186/s12981-022-00473-0)
Supplement: Supplementary file 1 — Supplementary Figures and Tables. [file 12981_2022_473_MOESM1_ESM.docx]

**SUPPLEMENT**

**Figure S1**. Directed acyclic graph for the relation between PrEP-related stigma and PrEP use.


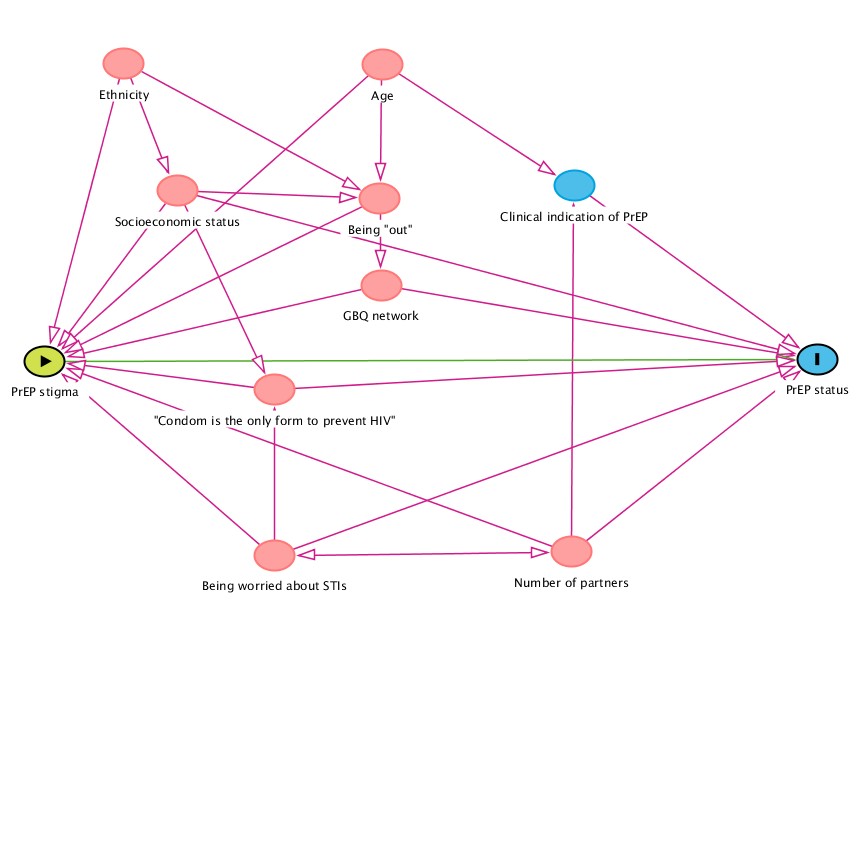


**Figure S2.** Distribution of scores for measuring stigma. The mean value was 6.77 (SD 1.66), the low stigma category corresponded to values below -1 SD, the intermediate category to values between -1 and +1 SD, and the high SD to values above +1 SD.

**Table S1.** Answers to individual statements about PrEP-related stigma by PrEP use.

|  | 1. **I think people who take PrEP are promiscuous*** | | | | | | | | | |
| --- | --- | --- | --- | --- | --- | --- | --- | --- | --- | --- |
|  | Strongly disagree (1 point) | | Disagree (2 points) | | Agree (3 points) | | Strongly agree (4 points) | | Prefers not to answer | |
| PrEP use | n | % | n | % | n | % | n | % | n | % |
| Never user | 117 | 20% | 229 | 39% | 159 | 27% | 47 | 8% | 37 | 6% |
| Former user | 26 | 13% | 82 | 41% | 64 | 32% | 21 | 11% | 5 | 3% |
| Current user | 116 | 23% | 225 | 45% | 112 | 22% | 37 | 7% | 12 | 2% |
| Total | 259 | 20% | 536 | 42% | 335 | 26% | 105 | 8.15 | 54 | 4% |
| *p-values: never vs current PrEP user: 0.126, former vs current: 0.002. | | | | | | | | | | |
|  |  |  |  |  |  |  |  |  |  |  |
|  | 1. **Other people think people who take PrEP are promiscuous*** | | | | | | | | | |
|  | Strongly disagree (1 point) | | Disagree (2 points) | | Agree (3 points) | | Strongly agree (4 points) | | Prefers not to answer | |
| PrEP use | n | % | n | % | n | % | n | % | n | % |
| Never user | 44 | 8% | 128 | 22% | 253 | 43% | 128 | 22% | 35 | 6% |
| Former user | 10 | 5% | 44 | 22% | 81 | 41% | 60 | 30% | 3 | 2% |
| Current user | 30 | 6% | 82 | 16% | 223 | 45% | 150 | 30% | 15 | 3% |
| Total | 84 | 7% | 254 | 20% | 557 | 43% | 338 | 26% | 53 | 4% |
| *p-values: never vs current PrEP user: 0.008, former vs current: 0.350. | | | | | | | | | | |
|  |  |  |  |  |  |  |  |  |  |  |
|  | 1. **I think people who take PrEP are responsible*** | | | | | | | | | |
|  | Strongly disagree (4 points) | | Disagree (3 points) | | Agree (2 points) | | Strongly agree (1 point) | | Prefers not to answer | |
| PrEP use | n | % | n | % | n | % | n | % | n | % |
| Never user | 22 | 4% | 46 | 8% | 241 | 41% | 256 | 43% | 25 | 4% |
| Former user | 8 | 4% | 22 | 11% | 80 | 40% | 84 | 42% | 4 | 2% |
| Current user | 14 | 3% | 13 | 3% | 147 | 29% | 310 | 62% | 17 | 3% |
| Total | 44 | 3% | 81 | 6% | 468 | 36% | 650 | 50% | 46 | 4% |
| *p-values: never vs current PrEP user: <0.001, former vs current: <0.001. | | | | | | | | | | |
